# Development of Novel Polyamide 11 Multifilaments and Fabric Structures Based on Industrial Lignin and Zinc Phosphinate as Flame Retardants

**DOI:** 10.3390/molecules25214963

**Published:** 2020-10-27

**Authors:** Neeraj Mandlekar, Aurélie Cayla, François Rault, Stéphane Giraud, Fabien Salaün, Jinping Guan

**Affiliations:** 1 GEMTEX-Laboratoire de Génie et Matériaux Textiles, ENSAIT, F-59000 Lille, France; neeraj.mandlekar@gmail.com (N.M.); aurelie.cayla@ensait.fr (A.C.); francois.rault@ensait.fr (F.R.); fabien.salaun@ensait.fr (F.S.); 2 Department of Applied Science and Technology, Politecnico di Torino, 15121 Alessandria, Italy; 3 College of Textile and Clothing Engineering, Soochow University, Suzhou 215123, China; guanjinping@suda.edu.cn

**Keywords:** industrial lignin, polyamide 11, zinc phosphinate, flame retardancy, biobased textile, thermal decomposition

## Abstract

Biobased lignin represents one of the possible materials for next-generation flame retardant additives due to its sustainability, environmental benefits and comparable efficiency to other flame retardant (FR) additives. In this context, this study presents the development of FR polyamide 11 (PA11) multifilament yarns and fabric structures containing different industrial lignins (i.e., lignosulfonate lignin (LL), and Kraft lignin (KL)) and zinc phosphinate (ZnP). The combination of ZnP and lignin (KL or LL) at different weight ratios were used to prepare flame retarded PA11 blends by melt mixing using a twin-screw extruder. These blends were transformed into continuous multifilament yarns by the melt-spinning process even at a high concentration of additives as 20 wt%. The mechanical test results showed that the combination of KL and ZnP achieved higher strength and filaments showed regularity in structure as compared to the LL and ZnP filaments. Thermogravimetric (TG) analysis showed the incorporation of lignin induces the initial decomposition (T_5%_) at a lower temperature; at the same time, maximum decomposition (T_max_) shifts to a higher temperature region and a higher amount of char residue is reported at the end of the test. Further, the TGA-FTIR study revealed that the ternary blends (i.e., the combination of LL or KL, ZnP, and PA11) released mainly the phosphinate compound, hydrocarbon species, and a small amount of phosphinic acid during the initial decomposition stage (T_5%_), while hydrocarbons, carbonyls, and phenolic compounds along with CO_2_ are released during main decomposition stage (T_max_). The analysis of decomposition products suggests the stronger bonds formation in the condensed phase and the obtainment of a stable char layer. Cone calorimetry exploited to study the fire behavior on sheet samples (polymer bulk) showed an improvement in flame retardant properties with increasing lignin content in blends and most enhanced results were found when 10 wt% of LL and ZnP were combined such as a reduction in heat release rate (HRR) up to 64% and total heat release (THR) up to 22%. Besides, tests carried out on knitted fabric structure showed less influence on HRR and THR but the noticeable effect on postponing the time to ignition (TTI) and reduction in the maximum average rate of heat emission (MARHE) value during combustion.

## 1. Introduction

Textiles play a significant role in our daily life. Most textile fibres and fabrics are made of petroleum-based thermoplastic polymers, which are flammable and present a potential fire risk. Flame retardant (FR) materials have been developed to reduce the risk either by inhibiting the possibility of the textile material ignition or reduction of the rate of heat release during flame spread. Halogen-based FR compounds, which have been in use since the 1930s, are still widely used and very efficient to enhance the fire-retardant behavior of textile materials. However, due to the persistency, bioaccumulation and toxicity of certain halogenated compounds to human health and stringent fire safety regulations, the scientific and industrial communities have been forced to find alternative sustainable solutions [1,2]. To this end there has been a strongly growing interest in sustainable resources, especially in the development of flame retardant systems made of biobased resources as environmentally-friendly non-halogenated alternatives [3,4,5].

In this context, lignin has gained attention as a bio-based additive in FR systems. Lignin is a naturally abundant polyphenolic compound that is obtained from lignocellulosic biomass. Industrial lignin is mainly produced as a by-product of the wood-pulping and paper-making industries. Lignosulphonate and Kraft lignin are the most commonly produced lignins on an industrial scale, which are isolated from the sulphite and the Kraft process, respectively. Owing to its highly aromatic framework, lignin achieves a very high char yield (around 40–50 wt%) upon thermal decomposition in an inert atmosphere [6]. It is reported that during the combustion the formation of the char layer is an important phenomenon to provide a good barrier against heat and oxygen diffusion in the polymer matrix [7,8,9]. Lignin has been employed as a carbon source in intumescent systems, i.e., in combination with other FR compounds in polypropylene [10], polylactic acid [11], polyurethane [12], and more recently with polyamide [13,14] matrices to provide flame retardancy. Recently, Cayla et al. studied the optimal ratio of lignin and ammonium polyphosphate (APP) combinations to develop FR polyethylene terephthalate (PET) multifilament yarns and fabric structures [15].

Polyamide 11 (PA11) is one of the most promising biobased thermoplastic engineering polymers, which is derived from a renewable resource (castor oil) [16]. Thanks to its excellent mechanical properties PA11 is used for various applications in many industries such as automotive, aerospace and sports [17,18]. Nowadays, PA11 is also exploited in the textile industries for making technical and high-end fabrics, brushes, filters and industrial woven fabrics [19,20]. However, the low flame retardant properties and extended dripping of PA11 limits its potential applications in high-end technical textiles. The effect of APP on the thermal stability of PA11 was studied by Levchik et al. [21]. It was suggested that the interaction of APP and PA11 forms the intermediate phosphate ester bonds which further decompose to favor the formation of an intumescent char. Some other research groups used the metal phosphinate and various types of nanoparticles (e.g., nanoclay, nanosilica and nanotubes) to improve the flame retardancy of PA11 [22,23,24]. Interaction between phosphinate FR and nanoparticles develops effective char layer in the condensed phase and flame retardancy was enhanced. As far as flame retardant PA11 fibres and textile is concerned, the most commonly used approach for the development of inherently fire-retardant polyamide fibres is melt mixing of compounds followed by a melt-spinning process. Usually, flame retardants that are commercially acceptable for engineering polymers, including PA11, require a high concentration (≥20 wt%) of FR additive in the polymer bulk [23,24,25,26]. However, most of the time a high concentration (>10 wt%) of FR is unsuitable for multifilament yarns due to the difficulties in spinning, causing clogging of filters and spinnerets [27,28] and a reduction in yarns’ tensile properties [29]. This problem has been overcome by the selection of FR additives which are miscible with the molten polymer matrix and optimization of the additive ratio in a polymer. For example, Didane et al. used the combination of ZnP and different polyhedral oligomeric silsesquioxanes (POSS) to obtain continuous multifilaments [30]. Recently, Vasiljevic et al. developed FR polyamide 6 (PA6)/bridged 9,10-dihydro-9-oxa-10-phosphaphenanthrene-10-oxide (DOPO)-derivative (PHED) nanocomposite textile filament yarns using 10 and 15 wt% of PHED [31].

In our earlier studies, industrial lignins were combined with zinc phosphinate FR to evaluate the flame retardant properties in PA11 blends. It was demonstrated that the combination of lignin and metal phosphinate is effective for the enhancement of flame retardancy attributed to the formation of a protective char layer in the condensed phase in polymer bulk [32,33]. Though flame retardancy of lignin with FR additives in polyamides has been investigated, the realization to obtained lignin-based FR multifilament yarns and textile structure has been unexplored. Hence, this present study investigates the development of multifilament yarns and fabric structure of PA11 blends based on industrial lignins (KL and LL) and zinc phosphinate FR. Different concentrations of lignin and ZnP up to 20 wt% were used to optimize the fire properties as well as to increase biobased character in the resulting textile structure. ZnP was chosen as an FR additive due to its miscibility with molten polymer, high phosphorus content (19.5–20.5 wt%), and thermal stability (>350 °C). The mechanical and physical properties of multifilament yarns were investigated. Besides, the analysis of thermal stability along with the analysis of evolved gas products was also studied to understand the decomposition behavior. The fire-retardant properties of the ternary blends as sheets (polymer bulk) were evaluated by the cone calorimeter test. Furthermore, the combustion behavior of knitted fabric structures was also assessed and compared with sheets sample.

## 2. Materials and Methods

### 2.1. Materials

A biobased Polyamide 11 (PA11), Rilsan^®^ BMNO-TLD; Mn = 17,000 g/mol, Melt flow index (MFI) = 14–20 g/10 min at 235 °C, supplied by Arkema (Colombes, France), was chosen as the polymer matrix for the realization of multifilament production. Zinc phosphinate (ZnP), Exolit^®^ OP 950, supplied by Clariant (Muttenz, Switzerland), was used as flame retardant. Two different types of industrial lignins were used as carbon source, varying in chemical nature: the first one is lignosulphonate lignin (coded as LL), provided from Domsjo Fabrikar AB (Örnsköldsvik, Sweden) and the second one is alkali kraft lignin (coded as KL) obtained from UPM Biochemicals, Helsinki, Finland (the European distributor of Domtar BioChoice^®^ lignin). LL contains mainly Na-lignosulfonate (about 70%), small amounts of Mg and Ca-lignosulfonates, and some impurities such as ash and carbohydrates (about 20%). KL comprises mostly alkali kraft with a 90% purity level. All the materials were dried at 80 °C for 12 h before use.

### 2.2. Blend Preparation and Processing

The different blend formulations based on PA11, ZnP, and two different lignins (LL and KL) were prepared by melt extrusion. A total of 20 wt% loading was fixed in all blend formulations; phosphinate was varied from 10 to 15 wt% and lignin from 5 to 10 wt%. All the formulations prepared for this study have been reported (Table S1, supporting file). These blends were prepared using a co-rotating intermeshing twin-screw extruder (PTW 16, L/D = 25, Thermo Haake, Waltham, MA, USA). The extruder has five heating zones and for this work, the temperature profiles of the five heating zones set as follows, 170, 190, 210, 210, and 220 °C, and the rotation speed was set at 100 rpm. The polymer in the form of granules and additives as powder form were mixed and introduced into the extruder at the feeding zone. The polymer was melted and mixed homogeneously with the additives by shearing, extruder output was then obtained in the form of a rod (1.5 mm). In all cases, the extruded rods were pelletized for further analysis. All the polymers and additives were dried at 80 °C for 12 h before processing and analysis. Sheet specimens (thickness of 3 mm) in accordance with cone calorimeter (100 × 100 mm^2^) were prepared by compression molding using a Teach-Line 200T hydraulic press (Collin Lab, Maitenbeth, Germany) operating at 60 bars and 220 °C.

### 2.3. Multifilament Development via Melt Spinning

Extruded blends were transformed into multifilament yarns using a pilot-scale melt spinning device (SPINBOY I, Busschaert Engineering, Outrijve, Belgium) as shown in Figure S1 (Supporting File). The extruder of the spinning device has six heating zones, five at the extruder and one at the spin-pack zone whose temperature profile ranged between 180 and 220 °C and spin-pack at 230 °C. Under thermal and mechanical stresses, pellets melt and are transported to the volumetric pump (chamber volume = 3.5 cm^3^) whose rotational speed was set 20 rpm to ensure a constant flow of molten polymer to the spinneret (80 holes, 1.2 mm diameter), giving consisted 80 filaments, which were cooled and coated with spin-oil to limit electrostatic effect. Multifilaments were then drawn between two rolls (supply roll (R_1_) and drawing out roll (R_2_), heated at 75 °C and 90 °C, respectively), with varying speeds S_1_ and S_2_ to ensure the drawing of multifilaments. The theoretical draw is given by the draw ratio DR = S_2_/S_1_ that applies a mechanical stretching on the multifilaments. Finally, the multifilaments were winded on a bobbin. The multifilaments produced for unfilled PA11 and PA-KL-ZnP blends achieved the stretching speed (S_2_) up to 200 m/min (DR = 2). However, the LL and ZnP blend found its drawing limit with a stretching speed (S_2_) up to 130 m/min (DR = 1.3), increasing this speed caused the disturbance and breakage in multifilament production.

### 2.4. Knitted Fabric Development

Knitted fabric structures were produced with the multifilaments to perform the cone calorimeter (fire behavior) tests. A double-layer structure of about 1400 ± 50 g/m^2^ and a thickness of around 3 mm was developed (Figure S2, supporting file) on a flat knitting machine (SES122ff, gauge E7, Shima Seiki, Wakayama, Japan). This structure was preferred due to its compactness and dimensional stability. It is important to note that knitted fabric samples were successfully developed for KL and ZnP combinations, however, multifilaments with LL and ZnP blends failed to produce fabric structures due to the lower mechanical strength of multifilament yarns.

### 2.5. Characterization Techniques

#### 2.5.1. Melt Flow Index

A Thermo Scientific Haake Melt Flow HT indexer compliant with the ASTM D1238 standard [34] was used for melt flow index (MFI) measurements to analyze spinning feasibility and to determine the spinning temperature parameters. Beforehand, the piston was preheated at 220 °C for 2 min, and then the barrel was filled with dried material (7 g per measurement) and heated for 4 min, and then measurements were carried out under a load of 2.16 kg. This procedure was repeated three times for each sample and the results were averaged.

#### 2.5.2. Optical Microscopy

Microscopic observations were performed by using a microscope (Axiolab, Carl Zeiss, Jena, Germany), equipped with a uEYE camera (IDS, Obersulm, Germany). All filled filaments of ternary blends were observed longitudinal and cross-section view at 20× magnification to analyze the mean diameter, the surface aspects, and regularity. Further, images were processed in ImageJ online available particle analysis software to measure the mean diameter.

#### 2.5.3. Mechanical Properties

Measurement of the mechanical properties of monofilament extracted from multifilaments was carried out on a model 1456 universal testing machine device (ZWICK, Ulm, Germany) following the ISO 5079 standard procedure. The force sensor of 10N was used. The length between jaws was 20 mm and the crosshead speed was 20 mm/min. Multiple tests, set of 10 was conducted using a standard conditioned sample at a confined temperature of 23 ± 2 °C and relative humidity (RH) of 65 ± 5%. Obtained results were then averaged and analyzed. At first, the maximum force at the breaking point was measured and then the elongation (%), elastic modulus (MPa), and tenacity (cN/Tex) were calculated from tensile testing data. Before mechanical experiments, the fineness of each monofilament was determined according to ISO 1973 using a vibroscope (Zweigle, Uster, Switzerland) which allows measuring the linear density of the monofilament in Tex (g/km).

#### 2.5.4. Thermal Decomposition

Thermogravimetric (TG) analyses were carried out with a Q500 thermal analyzer (TA Instruments, New Castle, DE, USA) in a nitrogen atmosphere at a purge rate of 60 mL/min, with a heating rate of 10 °C/min from 50 to 800 °C, using alumina pans and sample weight of 10 ± 0.2 mg. The decomposition parameters, such as onset of decomposition temperature at 5% weight loss (T_5%_), and residue at 700 °C were obtained from the TG curve. Subsequently, the maximum mass loss rate (MMLR) and the corresponding temperature (T_max_) were obtained from derivative curves (dTG). Furthermore, theoretical residue mass was calculated from the linear combination of individual residue mass and compared with the practical residue mass of the blend.

Alternatively, the analysis of evolved gas was carried out by TG-FTIR experiments. TGA-4000 thermo balance (Perkin Elmer, Shelton, CT, USA) coupled with an infrared spectrometer FTIR Spectrum Two (Perkin Elmer) was used to carry out these experiments in a nitrogen (flow 35 mL/min) atmosphere with a heating rate of 10 °C from 50 to 800 °C. In order to evaluate the gaseous decomposition products in real-time a transfer tube connecting the TG and the infrared cell was heated to 280 °C to avoid the condensation of the evolved gaseous products. The infrared spectrometer, equipped with a DTGS KBr detector, was operated in the range from 4000 to 500 cm^−1^ with 4 cm^−1^ optical resolution.

#### 2.5.5. Raman Spectroscopy

Raman spectroscopy of char residue of ternary blends was performed on ALMEGA Dispersive Raman spectrometer (Thermo Nicolet, Dreieich, Germany) with 514 nm excitation. The following wavelength was chosen to reduce noise level and yields a spectrum with a superior signal-to-noise ratio.

#### 2.5.6. Fire Behavior

Fire behavior was studied by cone calorimeter tests for sheet samples (polymer bulk) and fabric samples and compared by different flame retardant parameters. The oxygen consumption cone calorimeter was employed to assess the forced combustion behavior of the sheet sample (100 × 100 × 3 mm^3^) at a heat flux of 35 kW/m^2^ following ISO 5660 standard [35]. The distance between the sample and the heating cone was increased to 60 mm due to the material swelling behavior. Before performing the tests, all specimens were conditioned at 23 °C and 50 ± 3% RH for 72 h. Three tests were carried out on each formulation, and the results were averaged.

The measurements of knitted fabric samples were carried out on a fire testing technology (FTT) Mass Loss Calorimeter (MLC) instrument according to the ASTM E 906 standard procedure. To maintain dimensional stability of fabric samples a metallic grid was used during experiments. In principle, the HRR in MLC is calculated from thermal measure by a thermopile in the chimney rather than employing the oxygen consumption principle in the cone calorimeter. The adopted procedure involved exposing specimens (100 mm × 100 mm × 3 mm) in a horizontal orientation. The external heat flux of 35 kW/m^2^ used for these tests corresponds to common heat flux in a mild fire scenario. The data reported in this work are the average of three replicated experiments.

According to this test method, critical parameters, for example, time to ignition (TTI), heat release rate (HRR), total heat release (THR) were evaluated for sheet and fabric samples. Furthermore, from HRR the average rate of heat emission (ARHE) defined in Equation (1) and its maximum (MARHE) were calculated:

(1) $$ARHE(t_{n})=\frac{\sum_{2}^{n}(t_{n}-t_{n-1})\times \frac{\left(q_{n}-q_{n-1}\right)}{2}}{\left(t_{n}-t_{0}\right)}$$

where *t*_0_ is the time at the beginning of the test, *t_n_* is test time after *t*_0_, and *q_n_* is the rate of heat release at *t_n_*.

## 3. Results and Discussion

### 3.1. Melt Fluidity

**Figure 1:**
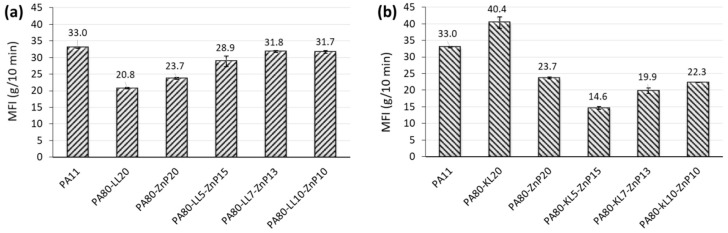
Melt flow index of PA11 and its blends with phosphinate and lignins: (**a**) PA-LL-ZnP blends, (**b**) PA-KL-ZnP blends.

The MFI of unfilled polymer and the blends has been evaluated, to have a qualitative assessment of the effect of lignin and phosphinate addition on the fluidity, and hence on the processing of the resulting blends. The trend of MFI as a function of the lignin and phosphinate content in the blends is shown in Figure 1a,b. The addition of 20 wt% of LL or ZnP in binary blends decreases the fluidity (MFI values). This reduction in MFI with LL 20 wt% is attributed to the presence of larger molecular chains (i.e., molecular weight) of sulphonated lignin and molecular weight distribution as observed by Kun and Pukánszky [36]. However, the combination of LL and ZnP within the ternary blends increases the fluidity with respect to neat PA11, plasticization effect of lignin is known in the literature [37]. Conversely, the introduction of KL in the binary blend increases the fluidity, which might be associated with the plasticization effect and the lower molecular weight of kraft lignin [36]. However, the combination of KL and ZnP reduces the MFI values, as the system turns out to reduce the fluidity; furthermore, the interactions between ZnP and KL contribute in achieving a good filler distribution [38]. Based on the MFI result it is observed that with slight variation in values the fluidity of the ternary blends remains in the range required for the spinning set up used in this study (i.e., between 15–30 g/10 min).

### 3.2. Structure and Surface Properties

**Figure 2:**
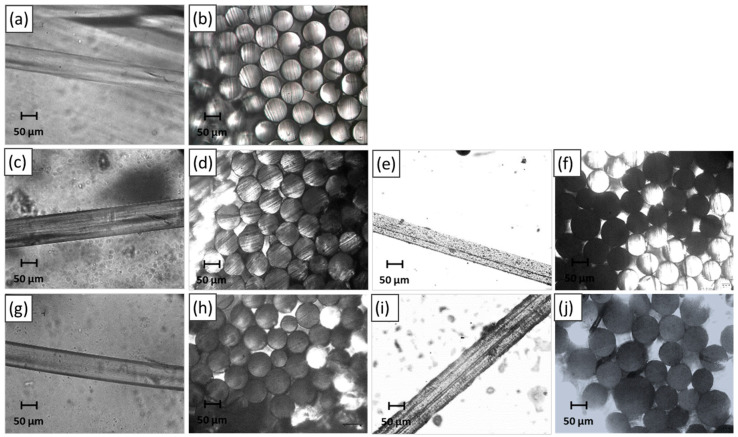
Microscopic images of surface and cross-section view of monofilament: (**a**,**b**) PA11; (**c**,**d**) PA80-KL5-ZnP15; (**e**,**f**) PA80-KL10-ZnP10; (**g**,**h**) PA80-LL5-ZnP15; (**i**,**j**) PA80-LL10-ZnP10.

**Figure 3:**
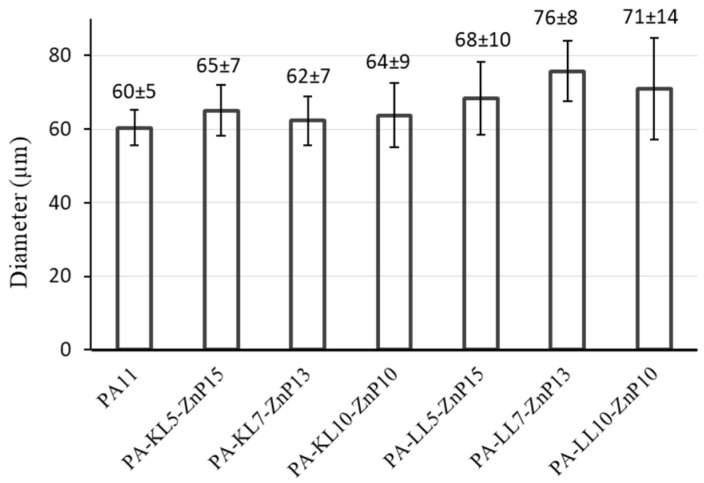
The average diameter of monofilaments extracted from multifilaments.

The microscopic images of the monofilament of PA11 and its blends filled with ZnP and lignin (KL or LL) are shown in Figure 2 to provide the qualitative observation of the surface smoothness and regularity of the fibres. Furthermore, the diameter of monofilament was also measured for at least 10 samples and averaged value has been reported (Figure 3). It is observed that PA11 has a smooth and regular surface with an average diameter of 60 ± 5 μm, showing uniform fibres diameter (Figure 2a,b). The ternary blends of PA-KL-ZnP report that the combination of KL and ZnP shows less effect on the surface irregularity of monofilament because the surface of the filament stays regular (Figure 2c,f) and the average size was between 62 and 65 μm (Figure 3). It is also found that KL and ZnP are well mixed and evenly dispersed in the polymer matrix, there was no visible agglomeration observed. Conversely, the monofilaments extracted from multifilament with LL and ZnP combination tend to have an irregular surface (Figure 2g–j) and the average size of monofilaments between 68 and 76 μm, which has a quite high variation in the diameter (Figure 3). This irregularity is related to the reduction of draw ratio and agglomeration (up to 10 μm) of particles on the surface. Larger the particle size create microvoids around the agglomerate, which under tension causes the propagation of microcracks, then coalescence and finally breakage of filament. As consequence, the spinnability of PA-LL-ZnP multifilaments was suffered and a limited draw ratio (DR = 1.3) was achieved compared to a DR = 2 for unfilled PA11 and PA-KL-ZnP multifilaments.

### 3.3. Mechanical Properties of Multifilament Yarns

**Figure 4:**
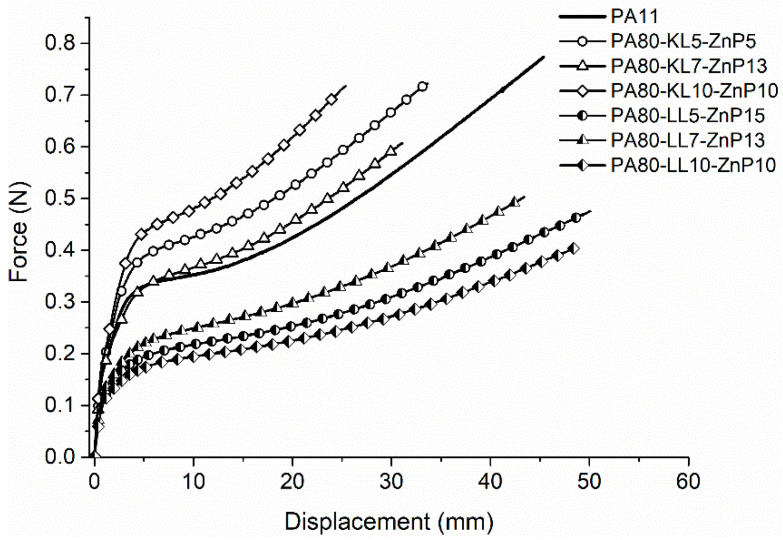
Force displacement curve for lignin and ZnP filled PA11 monofilaments.

The influence of ZnP and lignin addition on the mechanical properties of monofilaments was analyzed by tensile tests. Tensile properties such as modulus (MPa), force at break (cN), elongation (%) at break, and tenacity (cN/Tex) are reported in Table 1, and the force-displacement curve is shown in Figure 4. The fineness of monofilament yarns was also measured before testing of mechanical properties to evaluate the linear density (Figure S3, Supporting File). It is noticed that KL and ZnP combination gives consistent multifilament yarns with uniform linear density between 37 and 39 dTex, whereas LL and ZnP blends produce inconsistent yarns with considerable variation in linear density between 36 and 45 dTex. Moreover, variation increases with increasing the LL add-on and the blend with 10% LL has the highest variation. This behavior was attributed to the agglomerations of LL particles in the multifilaments which limits the spinning process.

PA11 monofilaments displayed force and elongation at break values of around 75 cN and 194%, and tenacity about 21.2 cN/Tex (Figure 4, Table 1). These properties were influenced by the addition of lignin and ZnP in PA11. The blends containing KL and ZnP resulted in a decrease in the % elongation and tenacity with increasing KL content from 5 to 10 wt% in ternary blends. In addition, elastic modulus increases with increasing KL and reducing ZnP content, which is attributed to better stress transfer between additive and polymer matrix. A higher modulus indicates the stiffness of filaments compared to unfilled PA11. On the other hand, filaments containing LL and ZnP show higher % elongation compared to that of unfilled PA11, at the same time other properties such as force at break, modulus, and tenacity are decreased significantly with increasing LL loading. This reduction in mechanical properties is noticed due to the uneven distribution and immiscibility of LL particles in the polymer matrix [33]. This behavior might be ascribed to the poor stress distribution from the matrix to LL. In our earlier study, it was found that LL has a larger particle size than KL in the original material [39]. As a consequence, tensile properties were degraded after the addition of LL in PA11.

Lower mechanical properties for PA-LL-ZnP multifilament also explain the obtainment of low draw ratio (DR = 1.3) and a larger diameter of these filaments. Consequently, LL and ZnP filled multifilament yarns were repeatedly broken during the twisting and yarn knitting process (fabric production). Therefore, LL and ZnP filaments failed to be transformed into the knitted fabric.

### 3.4. Thermal Properties

**Figure 5:**
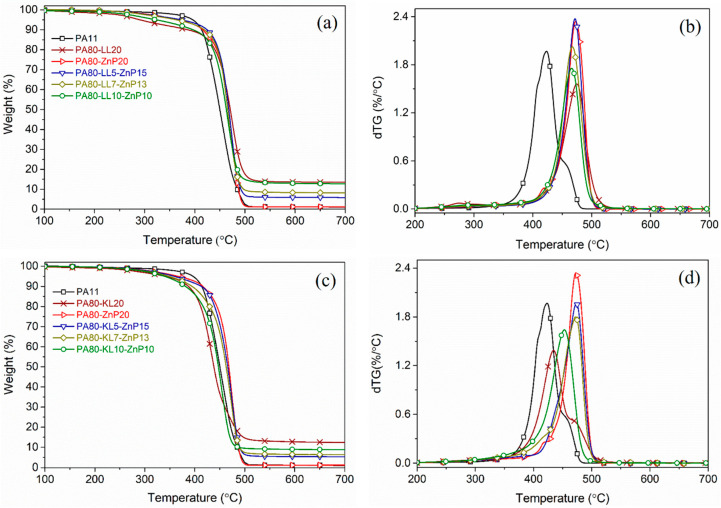
Thermograms for PA11, binary and ternary blends: (**a**,**b**) mass loss and derivative of weight loss curves for LL and ZnP blends, (**c**,**d**) mass loss and derivative of weight loss curves for KL and ZnP blends.

The influence of the incorporated lignin (KL or LL) and ZnP on the thermal decomposition behavior of PA11 blends was studied by TG analyses carried out under a nitrogen atmosphere. The TG data are summarized in Table 2 and the thermal degradation profile of unfilled PA11, binary, and ternary blends are shown in Figure 5. It is observed that decomposition of unfilled PA11 occurs mainly with single-step degradation taking place between 350 and 500 °C without leaving any considerable char residue at the end of the test. A sharp dTG profile indicates a rapid mass loss rate with a maximum decomposition temperature (T_max_) at 423 °C and a shoulder identified at around 450 °C is associated with cross-linked structure obtained during thermal decomposition [21].

The addition of LL or KL in binary blends initiates the degradation earlier (i.e., lowering T_5%_) at 285 and 341 °C, respectively. As compared to PA11, a significant reduction of T_5%_ temperature in LL blends was attributed to the decomposition of subunits present in LL and evolution of less thermally stable compounds, e.g., hydrocarbons and CO_2_ that leads to the formation of stronger bonds in the condensed phase and promotes the thermal stability [40]. LL blends shows T_max_ at a higher temperature region (468 °C) compared to the T_max_ (435 °C) of PA80-KL20 blend; consequently, the PA80-LL20 blend generates a higher amount of char residue (13.5 wt% at 700 °C) as compared to the calculated char residue 12.5 wt%. Besides, the thermal stability of binary blends of ZnP increases, and the decomposition step occurs around 460 °C. However, no residue is left from the PA80-ZnP20 blend [41].

In all ternary blends, the presence of KL or LL with ZnP shifted the initial decomposition temperature (T_5%_) to a lower temperature, at the same time T_max_ moved to a higher temperature range (Figure 5a,b). A decrease of T_5%_ of the ternary blends is ascribed to the lignin degradation, which starts at a lower temperature due to the scission of weak ether, aryl-alkyl, phenyl glycoside bonds, and unstable C-C bonds leading to the maximum evolution of decomposition products between 400 and 500 °C. In the meantime, structural rearrangement occurs in the condensed phase; as a consequence, a noticeable amount of char residue is collected at the end of the tests. It is noteworthy that increasing the LL loading content from 5 to 10 wt% in PA-LL-ZnP blends strongly reduces T_5%_ from 375 to 326 °C, respectively. The mass-loss rate is also decreased and T_max_ is shifted about 42 °C towards higher temperature region. Subsequently, these blends report higher char residue at the end of the test, for example, PA80-LL10-ZnP10 reported 12.7 wt% char residue at 700 °C with respect to 8.6 wt% calculated value. The presence of ZnP evolves phosphinate anion in the gas phase during decomposition and transformed into zinc phosphate [42], which also contributes to a thermally stable char residue. Hence, indicating the positive interaction between the additives and the polymer matrix. Moreover, it is previously reported that during the decomposition of LL, the sulphonate compounds release SO_2_ and transform into thermally stable Na_2_SO_4_, hence giving rise to the stable char. However, in this study evolution of SO_2_ is not detected by TGA-FTIR analysis. Shukla et al. also reported a similar finding [43].

Unlike PA-LL-ZnP blends, increasing KL content 5, 7, and 10 wt% in its ternary blends (Figure 5c,d), slightly influence the decrease of T_5%_ between 359 and 339 °C, is lower than T_5%_ of PA-LL-ZnP blends. On the other hand, T_max_ for PA80-KL5-ZnP15 and PA80-KL7-ZnP13 was similar to LL and ZnP blends and considerable change is noticed for PA80-KL10-ZnP10 where T_max_ shifted by 30 °C, leading to higher mass-loss between 400 and 500 °C. As a consequence, PA-KL-ZnP combination produces lower char residue compared to the theoretical value (calculated from the linear combination of individual residue mass) than that of PA-LL-ZnP blends.

### 3.5. Analysis of Released Products

**Figure 6:**
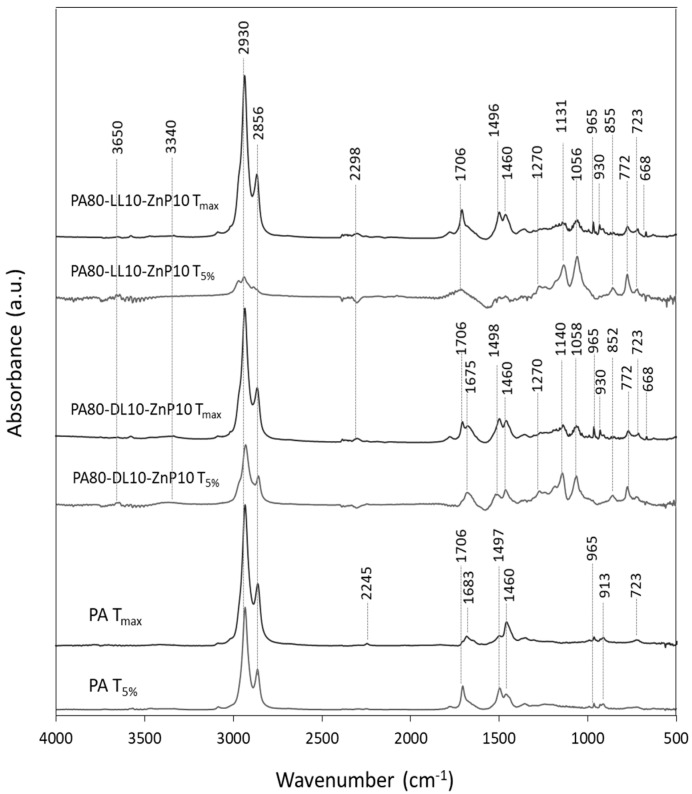
FTIR spectra of evolved gaseous products during thermal decomposition of PA11 and its blends with industrial lignin and ZnP.

The evolved gas products were also investigated by TG analyzer coupled with FTIR (TG-FTIR). The identification of released products and their characteristic peaks in FTIR spectra are reported in Table 3. The FTIR spectra of evolved gaseous products for T_5%_ and T_max_ decomposition temperature are shown in Figure 6. In PA11, FTIR spectrum of evolved gas products during initial decomposition temperature (T_5%_) shows the evolution hydrocarbons (2930, 2856, 723, 1460 cm^−1^), carbonyl compound (1706 cm^−1^), conjugated olefin (1497 cm^−1^), and a small amount of alkyl vinyl groups (1460, 965, 913 cm^−1^). During the main decomposition with the formation of nitrile (2245 cm^−1^), vinyl group, hydrocarbon species increases and carbonyl peak diminishes and amide group (1683 cm^−1^) is formed. Similar decomposition products are reported by other researchers [21,44]. The assignments of the detected FTIR bands for PA80-LL10-ZnP10 ternary blend at T_5%_ showed mainly the formation of phosphinate anion (1131, 1056, 772 cm^−1^) and a small amount of phosphinic acid (3650, 1270, 855 cm^−1^) due to the presence of ZnP and weak peak of hydrocarbon species (2930, 2856, 723 cm^−1^) and carbonyls (1706 cm^−1^) are related to the presence of LL. At T_max_, hydrocarbon species release increases and some additional characteristic peaks are observed, ascribing to carbonyl (1706 cm^−1^), phenolic compounds (1497, 3340 cm^−1^), and CO_2_ (2298, 668 cm^−1^). The ternary blend of KL and ZnP reveals the gas products release similar to that of LL and ZnP blends, the evolution of phosphinate anion (1140, 1058, 772 cm^−1^) and hydrocarbon species (2930, 2856, 1460, 723 cm^−1^) and phosphinic acid (3650, 1270, 855 cm^−1^) at the beginning (T_5%_) and carbonyls (1706 cm^−1^), phenolic compounds (1498, 3340 cm^−1^) and CO_2_ (2298, 668 cm^−1^) compounds are released during T_max_. The only difference relates to the intensity of hydrocarbons, carbonyls, and phenolic compounds are higher in PA-KL-ZnP which indicates that more volatile species are released at the beginning (T_5%_) of thermal decomposition of these blends.

### 3.6. Fire Behavior Analysis of Polymer Bulk

**Figure 7:**
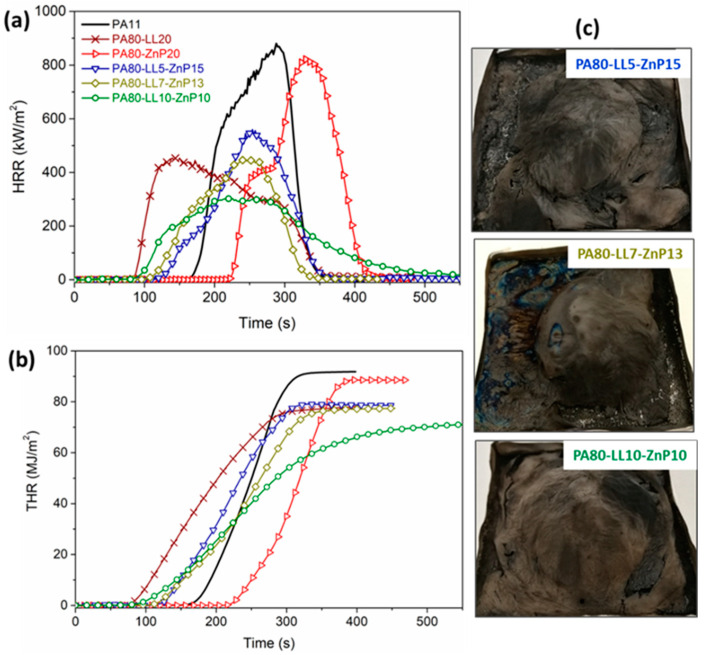
Cone calorimeter tests performed at 35 kW/m^2^ on polymer bulk (PA-LL-ZnP blends): (**a**) Heat release rate (HRR), (**b**) total heat release (THR) and (**c**) char residue images after combustion test.

**Figure 8:**
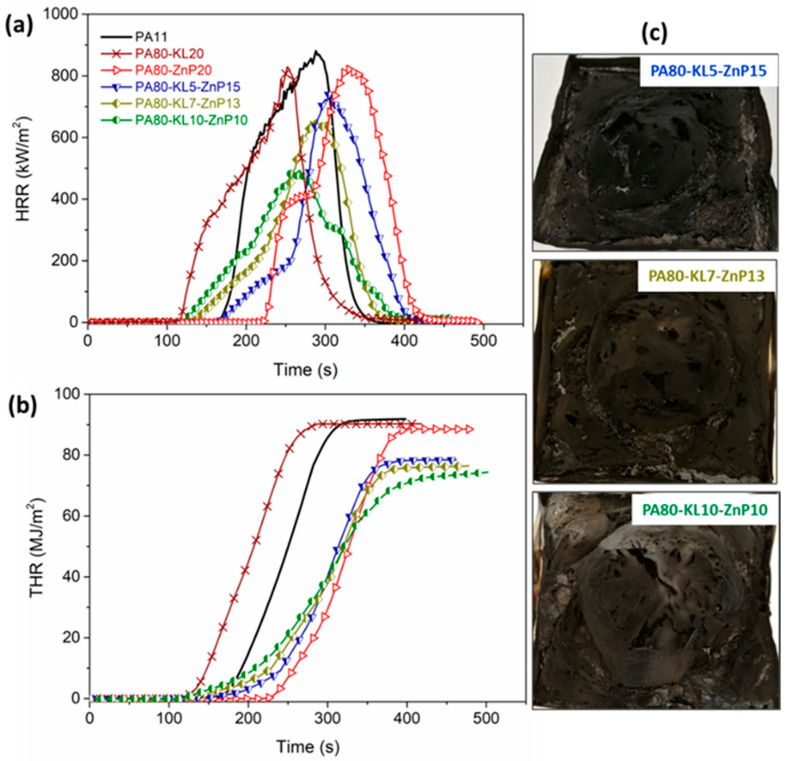
Cone calorimeter tests performed at 35 kW/m^2^ on polymer bulk (PA-KL-ZnP blends): (**a**) Heat release rate (HRR), (**b**) total heat release (THR), and (**c**) char residue images after combustion test.

Cone calorimeters are widely used to simulate the fire behavior of polymeric materials in a real fire scenario. The heat release rate (HRR) and total heat release (THR) curves are shown in Figure 7 and Figure 8. These potential fire hazards parameters are used to evaluate the combustion behavior of a material exposed under a certain heat flux [51,52]. Cone calorimeter data of PA11 and its blends characterized on sheet samples (polymer bulk) are reported in Table 4. As mentioned (Figure 7a,b) the addition of LL decreases TTI due to the rapid mass loss of LL taking place before the decomposition of PA11, which substantially decreases the peak of HRR from 884 to 454 kW/m^2^ and THR from 92 to 78 MJ/m^2^, respectively. The addition of ZnP (20 wt%) in binary blend shows delaying in TTI compared to that of unfilled PA11. After ignition at 223 s, the HRR for PA80-ZnP20 increases and the curve shows the thin char layer formation followed by its destruction, which is marked by the shoulder and followed by the continuous increase of PHRR up to 825 kW/m^2^. Furthermore, MARHE has reduced for LL or ZnP binary blends (about 22%) than unfilled PA11, showing some flame retardant effect. However, KL binary blend reported practically no effect in MARHE.

Besides, the ternary blends containing LL and ZnP showed lower TTI as compared to unfilled PA11. TTI value decreases with increasing LL content from 5 to 10 wt% in blends; this finding is attributed to the presence of LL. Besides, PHRR and THR values substantially drop (by 64% and 22%, respectively) for the PA80-LL10-ZnP10 blend, due to the formation of a protective char layer in the condensed phase that can effectively reduce the heat release during combustion. MARHE values were also decreased between 32 and 49% for these blends compared to unfilled PA11. It is assumed that LL contains sulfonate compounds, which are likely to decompose during combustion and produce thermally stable Na_2_SO_4_ in the condensed phase [53]. This enhanced fire-retardant properties in forced combustion tests are ascribed to the presence of LL and ZnP, which are able to confer stability to the char residue by the formation of a barrier layer. The increased char residue at the end of the test further confirms the formation of the protective layer. Hence, these results demonstrate that the proposed LL/ZnP combinations are effective.

It is noteworthy that unlike LL, the addition of KL in binary blends increases the HRR and its peak showing only a marginal reduction in PHRR with 5 and 7% KL loading (Figure 8a,b). The combination of KL and ZnP (ternary blends) decreases the TTI with increasing KL loading; for instance, PA80-KL5-ZnP15 blend ignites at 150 s close to PA11 whereas PA80-KL10-ZnP10 reports TTI at 116 s. MARHE value for these blends also reduced between 42% and 55% with increasing KL content 5 to 10 wt%.

Moreover, PHRR and THR values decrease slowly with the addition of KL in blends; a certain reduction in PHRR (−43%) is seen when KL content achieves 10 wt%. Higher PHRR in KL containing blends is linked with the higher mass loss of chemical subunits in KL, which shows maximum decomposition during combustion, similar behavior with kraft lignin and PA11 blends have been reported in our previous study [39], As a consequence, a less effective char layer is formed to reduce the HRR and its peak values.

**Figure 9:**
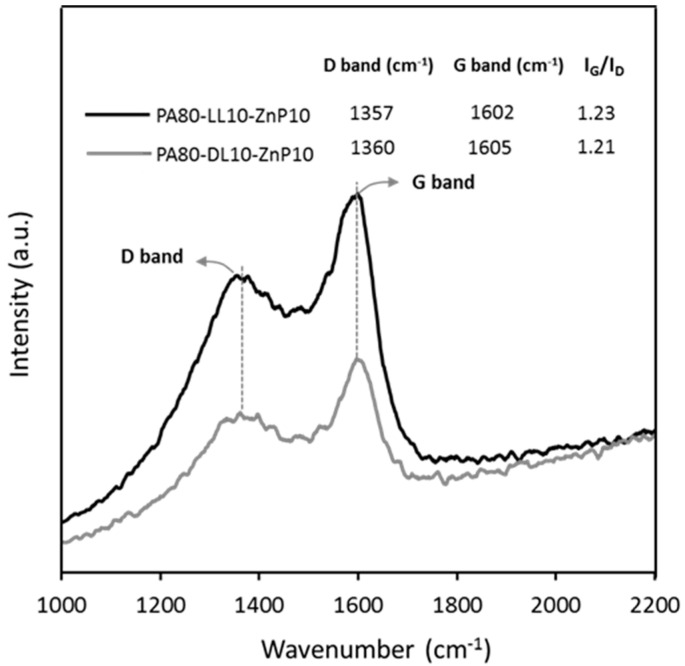
Raman spectra pattern of char residue was collected from the combustion test for PA80-LL10-ZnP and PA80-KL10-ZnP10 ternary blends.

To verify the presence of graphitic structure and to determine the hybridization state of carbon atoms in the residue char, Raman spectroscopy is conducted for ternary blends, namely, PA80-LL10-ZnP10 and PA80-KL10-ZnP10, as shown in Figure 9. Two strong peaks are observed at 1357 cm^−1^ and 1360 cm^−1^, which belong to the D band and peaks at 1602 cm^−1^ and 1605 cm^−1^ belong to the G band, respectively. Generally, the G band indicates the presence of crystalline graphitic carbon, sp^2^ hybrid carbon, in the char residue, while the D band derives from the defects of long-ranged disorder in the graphitic layer, sp^3^ hybrid carbon and other impurities [54]. This suggests that there are graphitic and disordered carbon structures coexisting in the char residue. The appearance of these two peaks demonstrates that the char residue is a highly conjugated polyaromatic structure [55]. In addition, the relative intensity ratio (R), namely I_G_/I_D_, allowed to evaluate the degree of graphitization of the char, and the smaller the value of I_G_/I_D_, the higher the degree of graphitization [56]. It was found that the I_G_/I_D_ value of PA80-LL10-ZnP10 char 1.23 is slightly higher than that of PA80-KL10-ZnP10 char 1.21, respectively, which means the degree of graphitization of PA80-KL10-ZnP10 char residue is slightly increased. This illustrated that the char has partially graphitic or highly conjugated polyaromatic structure, working as a protective layer. However, cone calorimeter results exhibit that the PA80-LL10-ZnP10 blend achieved greater flame retardant performance because of better resistance of char with higher barrier properties owing to the effective interaction of LL and ZnP.

### 3.7. Fire Behavior Analysis of Fabric Sample

**Figure 10:**
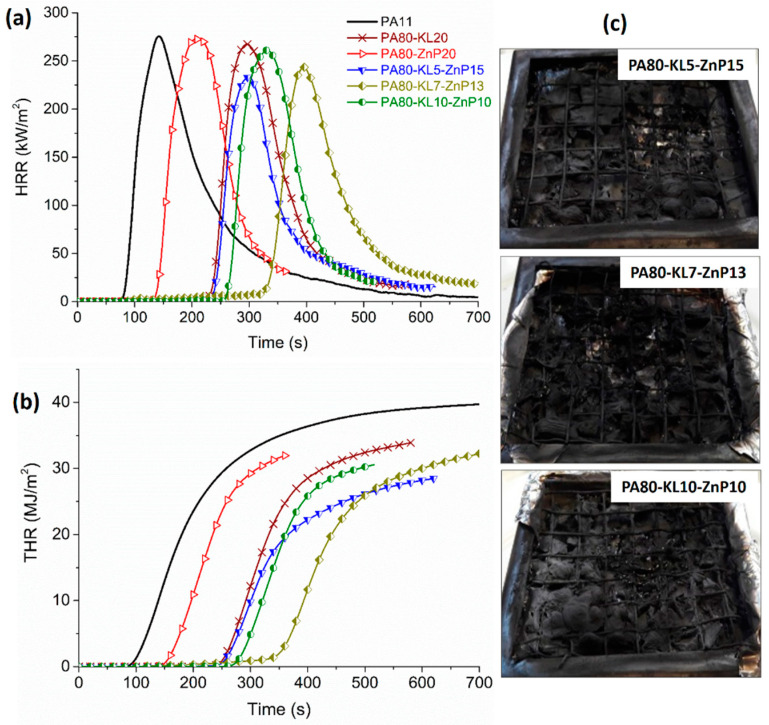
Cone calorimeter tests performed at 35 kW/m^2^ on fabric samples (PA-KL-ZnP blends): (**a**) Heat release rate (HRR), (**b**) total heat release (THR), and (**c**) char residue images after combustion test.

Cone calorimeter tests on knitted fabric samples were performed using a 35 kW/m^2^ heat flux which simulates a mild fire exposure and it is especially used for textile materials. It is noteworthy that when fire properties were evaluated on fabrics produced from KL and ZnP-filled multifilaments. LL and ZnP-based multifilaments were failed to incorporate into the fabric structure. HRR and THR curves with respect to time for fabric samples are shown in Figure 10a,b. Cone calorimetry data are collected in Table 5. It has been observed that the PHRR and THR are less influenced as compared to that of the unfilled PA11 fabric sample, similar behavior is reported in the literature for different fabric material, wherein HRR and THR are slightly changed [31,57]. The PA11 fabric sample quickly ignites after 78 s, rapidly increases the HRR and reaches PHRR about 278 kW/m^2^, and then decreases with the total heat released of around 38 MJ/m^2^. Besides, in PA80-ZnP20 fabric, the presence of ZnP slightly delays the ignition time to 122 s, whereas the fabric sample of PA80-KL20 shows ignition (TTI) after 184 s, reaching PHRR about 246 kW/m^2^ and the flame extinguishes in 265 s. No significant reduction in the value of HRR and PHRR is observed, indicating that the char layer formed in the condensed phase is not capable of reducing the evolution of combustible products. Similar behavior with kraft lignin and PLA fabric sample has been reported wherein the HRR and PHRR were not significantly reduced with the lignin addition [15].

All the combinations of KL and ZnP showed delayed TTI as compared to that of neat PA11, which is attributed to delay in the formation of an effective gas mixture that can trigger the ignition. In addition, the HRR and PHRR are slightly reduced; at the same time, THR and MARHE greatly influenced. For example, the PA80-KL5-ZnP15 sample shows the PHRR reduction of about 16% and THR around 21% than that of PA11, besides, increasing the lignin content in PA80-KL7-ZnP13 significantly reduces the MARHE up to 54%. This combination gives better flame retardancy compared to the other two formulations. Increasing char residue is observed due to the presence of lignin showing the charring effect. Furthermore, a visually higher amount of char residue is observed with increasing lignin content.

Based on the fire behavior testing of polymer bulk and fabric samples it is concluded that ignition and burning rate during forced combustion tests are different in polymer bulk and fabric structure under test conditions. Fibres have different geometry and the response to the fire would be different from polymer bulk. A general decrease in heat release parameters value (HRR, THR, and MARHE) is observed for fabric samples than polymer bulk due to the amount of polymer available as fuel in fabric for combustion is less in fabric samples. Ignition time in the fabric is higher than polymer bulk because volatile leaving from the heated sample and the oxygen takes more time to form an effective gaseous mixture that reaches to critical heat flux and starts the ignition. In sheet samples, PA-LL-ZnP blends offer better flame retardant properties than PA-KL-ZnP blends. However, PA-LL-ZnP multifilaments failed to produce fabric structures due to their lower mechanical properties. Fabric produced from the PA-KL-ZnP combination showed the best flame retardant performance for PA80-KL7-ZnP13.

## 4. Conclusions

In this study, two different industrial lignins (KL and LL) have been explored as char former in combination with zinc phosphinate FR additive and blended with PA11 polymer matrix at a higher concentration of 20 wt% by varying the additive ratio. The ternary blends containing industrial lignin (KL or LL) and ZnP were transformed into multifilament yarns by melt spinning process and then knitted fabric structures were developed. Tensile testing results have shown the mechanical properties of PA-LL-ZnP multifilaments were lowered than PA-KL-ZnP due to poor dispersion of LL particles in the PA11 matrix that has explained the failure of getting fabric structures for these blends. TG analysis showed the incorporation of lignin promotes the initial degradation (T_5%_) to begin at a lower temperature due to the presence of weak bonds in lignin, which led to the structural rearrangement and the formation of stronger bonds in the condensed phase; as result, the thermal stability of PA11 blends is increased. Further, the TG-FTIR study revealed that the ternary blends released phosphinate compound and phosphinic acid along with the hydrocarbon species, carbonyls and phenolic compounds, and CO_2_ during thermal decomposition. It was noticed that phosphinic acid was released only with the initial decomposition step (T_5%_). Forced combustion tests on polymer bulk have demonstrated that the interaction between lignin (KL or LL) and ZnP promoted a reduction in PHRR, THR and MARHE. In particular, the best flame retardant performance was found for LL and ZnP combination (i.e., PA80-LL10-ZnP10), resulting in a strong reduction in PHRR (−64%), THR (−22%) and MARHE (−49%) values. The formation of zinc phosphate and sodium sulphate compound in the condensed phase due to the presence of LL and ZnP was responsible for the protective char layer formation, which improves the fire-retardant properties. Besides, the cone calorimeter tests of the PA-KL-ZnP fabric sample showed a small reduction in HRR and PHRR value, which was expected due to the presence of less polymer amount as fuel. However, the presence of lignin significantly influenced the TTI and MARHE due to the char residue formation showing the charring effect. Among these formulations, the best fire-retardant properties were found with the PA80-KL7-ZnP13 fabric sample. From the overall point of view, this combination of industrial lignin and ZnP FR is found attractive due to the high availability and low cost of lignin and environmentally friendly FR alternative like ZnP. This work provides a cost-effective and sustainable approach towards the development of fully biobased textile FR textiles.

## Figures and Tables

**Table 1:** Tensile properties of multifilament yarns.

Sample	Elastic Modulus (MPa)	Force at Break (cN)	Elongation (%)	Tenacity (cN/Tex)
PA11	1.22 ± 0.20	75 ± 11	194 ± 32	21.2 ± 2.3
PA80-KL5-ZnP15	1.28 ± 0.20	77 ± 5	173 ± 15	19.8 ± 1.7
PA80-KL7-ZnP13	1.12 ± 0.13	57 ± 9	158 ± 15	16.1 ± 1.5
PA80-KL10-ZnP10	1.68 ± 0.14	64 ± 7	122 ± 13	18.5 ± 1.5
PA80-LL5-ZnP15	0.57 ± 0.20	47 ± 6	253 ± 37	12.7 ± 2.2
PA80-LL7-ZnP13	0.81 ± 0.20	51 ± 6	178 ± 61	12.4 ± 1.7
PA80-LL10-ZnP10	0.60 ± 0.29	39 ± 8	246 ± 65	12.0 ± 4.2

**Table 2:** Thermogravimetric data for PA11 and its blends with lignins and ZnP.

Sample	T_5%_ (°C)	T_max_ (°C)	MMLR (%/°C)	R_exp_@700 (%)	R_cal_ (%)
PA11	396	423	2.0	0.7	-
PA80-LL20	285	468	1.6	13.5	12.5
PA80-KL20	341	435	1.4	12.4	9.1
PA80-ZnP20	366	473	2.3	1.2	4.8
PA80-LL5-ZnP15	373	472	2.4	5.8	6.7
PA80-LL7-ZnP13	364	467	2.0	8.2	7.5
PA80-LL10-ZnP10	326	465	1.8	12.7	8.6
PA80-KL5-ZnP15	359	473	2.0	5.4	5.8
PA80-KL7-ZnP13	347	472	1.8	6.4	6.3
PA80-KL10-ZnP10	339	453	1.6	8.9	6.9

R_exp_ = experimentally recorded residue; R_cal_ = theoretically calculated residue.

**Table 3:** Main decomposition products and their characteristic IR frequency [41,45,46,47,48,49,50].

Decomposition Products	Functional Group	Characteristic Peak (cm^−1^)
**Polyamide 11**		
Hydrocarbon species (R-CH_2_-CH_3_)	-C-H_2_-	2930, 2856, 1460, 723
Alkyl vinyl (R-CH=CH_2_)	-CH=CH_2_ (cis-substitution)	1460, 965, 913
Olefin (conjugated)	-C=C-	1497
Carbonyl compound	>C=O	1706
Alkyl nitrile	-C≡N	2245
Amide compound	-CO-NH_2_-	1683
**PA80-KL10-ZnP10**		
Phosphinate compound	P=O, P-O	1140, 1058, 772
Phosphinic acid	P-OH	3650, 1270, 855
Hydrocarbon species (-CH_2_-, CH_3_-)	C-H	2930, 2856, 1460, 723
Phenolic compound	-C=C-, -OH	1498, 3340
Carbonyl compound	C=O	1706, 1675 (conjugation)
Carbon dioxide	O=C=O	2298, 668
**PA80-LL10-ZnP10**		
Phosphinate compound	P=O, P-O	1131, 1056, 772
Hydrocarbon species (-CH_2_-, CH_3_-)	C-H	2930, 2856, 1460, 723
Phosphinic acid	P-OH	3650, 1270, 855
Carbonyl compound	C=O	1706
Phenolic compound	-C=C-, -OH	1496, 3340
Carbon dioxide	O=C=O	2298, 668

**Table 4:** Cone calorimetry data for PA11 and its blends.

Sample	TTI (s)	∆ (%)	PHRR (kW/m^2^)	∆ (%)	THR (MJ/m^2^)	∆ (%)	MARHE (kW/m^2^)	∆ (%)
PA11	154 ± 3	-	884 ± 4	-	92 ± 4	-	281 ± 2	-
PA80-LL20	72 ± 12	−53	454 ± 30	−49	78 ± 6	−15	218 ± 5	−22
PA80-KL20	112 ± 10	−27	821 ± 27	−7	90 ± 2	−2	284 ± 3	+1
PA80-ZnP20	223 ± 14	+31	825 ± 29	−7	88 ± 6	−4	220 ± 4	−22
PA80-LL5-ZnP15	112 ± 12	−27	560 ± 40	−37	79 ± 2	−14	192 ± 20	−32
PA80-LL7-ZnP13	92 ± 9	−40	443 ± 21	−50	77 ± 4	−16	167 ± 7	−41
PA80-LL10-ZnP10	86 ± 9	−44	315 ± 11	−64	73 ± 2	−21	143 ± 14	−49
PA80-KL5-ZnP15	150 ± 18	−3	740 ± 23	−16	79 ± 2	−14	163 ± 11	−42
PA80-KL7-ZnP13	128 ± 14	−17	678 ± 36	−23	77 ± 3	−16	148 ± 15	−47
PA80-KL10-ZnP10	116 ± 13	−25	500 ± 48	−43	75 ± 7	−18	127 ± 18	−55

**Table 5:** Cone calorimeter data for knitted fabric structures.

Sample	TTI (s)	∆ (%)	PHRR (kW/m^2^)	∆ (%)	THR (MJ/m^2^)	∆ (%)	MARHE (kW/m^2^)	∆ (%)
PA11	78 ± 5	-	278 ± 2	-	38 ± 4	-	114 ± 4	-
PA80-KL20	184 ± 68	+58	246 ± 30	−12	31 ± 4	−17	75 ± 3	−34
PA80-ZnP20	122 ± 21	+36	287 ± 19	+3	32 ± 2	−15	106 ± 5	−7
PA80-KL5-ZnP15	209 ± 40	+63	234 ± 1	−16	30 ± 1	−21	66 ± 7	−43
PA80-KL7-ZnP13	294 ± 42	+73	237 ± 8	−15	32 ± 4	−16	52 ± 0	−54
PA80-KL10-ZnP10	198 ± 90	+61	247 ± 20	−11	31 ± 0	−17	75 ± 7	−34

## Supplementary Materials

The following are available online. Figure S1: Schematic of melt spinning machine; Figure S2: KL and ZnP filled PA11 knitted fabric structure; Figure S3: The linear density of monofilament yarns; Table S1: Prepared blend formulations.

## References

[1] Birnbaum L.S., Staskal D.F. (2004). Brominated flame retardants: Cause for concern?. Environ. Health Perspect..

[2] Darnerud P.O. (2003). Toxic effects of brominated flame retardants in man and in wildlife. Environ. Int..

[3] Cheng X.-W., Guan J.-P., Tang R.-C., Liu K.-Q. (2016). Phytic acid as a bio-based phosphorus flame retardant for poly(lactic acid) nonwoven fabric. J. Clean. Prod..

[4] Feng Y., Zhou Y., Li D., He S., Zhang F., Zhang G. (2017). A plant-based reactive ammonium phytate for use as a flame-retardant for cotton fabric. Carbohydr. Polym..

[5] Morgan A.B., Wilkie C.A. (2014). Non-Halogenated Flame Retardant Handbook.

[6] Li B., Zhang X., Su R. (2002). An investigation of thermal degradation and charring of larch lignin in the condensed phase: The effects of boric acid, guanyl urea phosphate, ammonium dihydrogen phosphate and ammonium polyphosphate. Polym. Degrad. Stab..

[7] Bertini F., Canetti M., Cacciamani A., Elegir G., Orlandi M., Zoia L. (2012). Effect of ligno-derivatives on thermal properties and degradation behavior of poly(3-hydroxybutyrate)-based biocomposites. Polym. Degrad. Stab..

[8] Canetti M., Bertini F., De Chirico A., Audisio G. (2006). Thermal degradation behaviour of isotactic polypropylene blended with lignin. Polym. Degrad. Stab..

[9] Song P., Cao Z., Fu S., Fang Z., Wu Q., Ye J. (2011). Thermal degradation and flame retardancy properties of ABS/lignin: Effects of lignin content and reactive compatibilization. Thermochim. Acta.

[10] De Chirico A., Armanini M., Chini P., Cioccolo G., Provasoli F., Audisio G. (2003). Flame retardants for polypropylene based on lignin. Polym. Degrad. Stab..

[11] Réti C., Casetta M., Duquesne S., Bourbigot S., Delobel R. (2008). Flammability properties of intumescent PLA including starch and lignin. Polym. Adv. Technol..

[12] Lu W., Li Q., Zhang Y., Yu H., Hirose S., Hatakeyama H., Matsumoto Y. (2018). Lignosulfonate/APP IFR and its flame retardancy in lignosulfonate-based rigid polyurethane foams. J. Wood Sci..

[13] Cayla A., Rault F., Giraud S., Salaün F., Sonnier R., Dumazert L. (2019). Influence of Ammonium Polyphosphate/Lignin Ratio on Thermal and Fire Behavior of Biobased Thermoplastic: The Case of Polyamide 11. Materials.

[14] Kundu C.K., Li Z., Li X., Zhang Z., Hu Y. (2020). Graphene oxide functionalized biomolecules for improved flame retardancy of Polyamide 66 fabrics with intact physical properties. Int. J. Biol. Macromol..

[15] Cayla A., Rault F., Giraud S., Salaün F., Fierro V., Celzard A. (2016). PLA with intumescent system containing lignin and ammonium polyphosphate for flame retardant textile. Polymers.

[16] Arkema Inc. (2012). Rilsan^®^ PA11: Created from a Renewable Source. https://www.researchgate.net/file.PostFileLoader.html?id=56bf97be7eddd380108b458d&assetKey=AS%3A328772155920384%401455396798180.

[17] Da Costa A.P., Botelho E.C., Costa M.L., Narita N.E., Tarpani J.R. (2012). A Review of Welding Technologies for Thermoplastic Composites in Aerospace Applications. J. Aerosp. Technol. Manag..

[18] Holbery J., Houston D. (2006). Natural-fiber-reinforced polymer composites in automotive applications. JOM.

[19] Cook J. (2004). Handbook of Textile Fibres. Natural Fibres.

[20] Wesołowski J., Płachta K. (2016). The Polyamide Market. Fibres Text. East. Eur..

[21] Levchik S.V., Costa L., Camino G. (1992). Effect of the fire-retardant, ammonium polyphosphate, on the thermal decomposition of aliphatic polyamides. I. Polyamides 11 and 12. Polym. Degrad. Stab..

[22] Jin X., Chen C., Sun J., Zhang X., Gu X., Zhang S. (2017). The synergism between melamine and expandable graphite on improving the flame retardancy of polyamide 11. High Perform. Polym..

[23] Hao A., Wong I., Wu H., Lisco B., Ong B., Sallean A., Butler S., Londa M., Koo J.H. (2015). Mechanical, thermal, and flame-retardant performance of polyamide 11–halloysite nanotube nanocomposites. J. Mater. Sci..

[24] Lao S.C., Koo J.H., Moon T.J., Londa M., Ibeh C.C., Wissler G.E., Pilato L.A. (2011). Flame-retardant polyamide 11 nanocomposites: Further thermal and flammability studies. J. Fire Sci..

[25] Hou W., Fu Y., Zeng C., Liu N., Yin C. (2020). Enhancement of flame retardancy and mechanical properties of polyamide 6 by incorporating melamine cyanurate combined with attapulgite. J. Appl. Polym. Sci..

[26] Lao S.C., Koo J.H., Moon T.J., Londa M., Ibeh C.C., Wissler G.E., Pilato L.A. (2009). Flame-retardant Polyamide 11 and 12 nanocomposites: Thermal and Flammability Properties. J. Compos. Mater..

[27] Bourbigot S., Flambard X. (2002). Heat Resistance and Flammability of High Performance Fibres: A Review. Fire Mater..

[28] Horrocks R., Sitpalan A., Zhou C., Kandola B.K. (2016). Flame Retardant Polyamide Fibres: The Challenge of Minimising Flame Retardant Additive Contents with Added Nanoclays. Polymers.

[29] Weil E.D., Levchik S.V. (2016). Flame Retardants for Plastics and Textiles.

[30] Didane N., Giraud S., Devaux E., Lemort G. (2012). A comparative study of POSS as synergists with zinc phosphinates for PET fire retardancy. Polym. Degrad. Stab..

[31] Vasiljević J., Čolović M., Jerman I., Simončič B., Demšar A., Samaki Y., Šobak M., Šest E., Golja B., Leskovšek M. (2019). In situ prepared polyamide 6/DOPO-derivative nanocomposite for melt-spinning of flame retardant textile filaments. Polym. Degrad. Stab..

[32] Mandlekar N., Malucelli G., Cayla A., Rault F., Giraud S., Salaün F., Guan J. (2018). Fire retardant action of zinc phosphinate and polyamide 11 blend containing lignin as a carbon source. Polym. Degrad. Stab..

[33] Mandlekar N., Cayla A., Rault F., Giraud S., Salaün F., Guan J. (2019). Valorization of Industrial Lignin as Biobased Carbon Source in Fire Retardant System for Polyamide 11 Blends. Polymers.

[34] ASTM (2013). D1238-13 Standard Test Method for Melt Flow Rates of Thermoplastics by Extrusion Plastometer.

[35] ISO (2002). ISO 5660-1:2002-Reaction-to-Fire Tests--Heat Release, Smoke Production and Mass Loss Rate--Part 1: Heat Release Rate (Cone Calorimeter Method).

[36] Kun D., Pukánszky B. (2017). Polymer/lignin blends: Interactions, properties, applications. Eur. Polym. J..

[37] Bouajila J., Dole P., Joly C., Limare A. (2006). Some laws of a lignin plasticization. J. Appl. Polym. Sci..

[38] Sallem-Idrissi N., Van Velthem P., Sclavons M. (2018). Fully Bio-Sourced Nylon 11/Raw Lignin Composites: Thermal and Mechanical Performances. J. Polym. Environ..

[39] Mandlekar N., Cayla A., Rault F., Giraud S., Salaün F., Malucelli G., Guan J. (2017). Thermal Stability and Fire Retardant Properties of Polyamide 11 Microcomposites Containing Different Lignins. Ind. Eng. Chem. Res..

[40] Nassar M.M., MacKay G.D.M. (1984). Mechanism of thermal decomposition of lignin. Wood Fiber Sci..

[41] Braun U., Schartel B. (2008). Flame Retardancy Mechanisms of Aluminium Phosphinate in Combination with Melamine Cyanurate in Glass-Fibre-Reinforced Poly(1,4-butylene terephthalate). Macromol. Mater. Eng..

[42] Vannier A., Duquesne S., Bourbigot S., Alongi J., Camino G., Delobel R. (2009). Investigation of the thermal degradation of PET, zinc phosphinate, OMPOSS and their blends—Identification of the formed species. Thermochim. Acta.

[43] Shukla A., Sharma V., Basak S., Ali S.W. (2019). Sodium lignin sulfonate: A bio-macromolecule for making fire retardant cotton fabric. Cellulose.

[44] Mailhos-Lefievre V., Sallet D., Martel B. (1989). Thermal degradation of pure and flame-retarded polyamides 11 and 12. Polym. Degrad. Stab..

[45] Braun U., Bahr H., Sturm H., Schartel B. (2008). Flame retardancy mechanisms of metal phosphinates in combination with melamine cyanurate in glass-fiber reinforced poly (1,4-butylene terephthalate): The influence of metal cation. Polym. Adv. Technol..

[46] Brebu M., Tamminen T., Spiridon I. (2013). Thermal degradation of various lignins by TG-MS/FTIR and Py-GC-MS. J. Anal. Appl. Pyrolysis.

[47] Levchik S.V., Costa L., Camino G. (1994). Effect of the fire-retardant ammonium polyphosphate on the thermal decomposition of aliphatic polyamides. Part III-Polyamides 6.6 and 6.10. Polym. Degrad. Stab..

[48] Naik A.D., Fontaine G., Samyn F., Delva X., Louisy J., Bellayer S., Bourgeois Y., Bourbigot S. (2014). Outlining the mechanism of flame retardancy in polyamide 66 blended with melamine-poly(zinc phosphate). Fire Saf. J..

[49] Socrates G. (2001). Infrared and Raman Characteristic Group Frequencies Contents.

[50] Zhao J., Xiuwen W., Hu J., Liu Q., Shen D., Xiao R. (2014). Thermal degradation of softwood lignin and hardwood lignin by TG-FTIR and Py-GC/MS. Polym. Degrad. Stab..

[51] Mouritz A.P., Mathys Z., Gibson A.G. (2006). Heat release of polymer composites in fire. Compos. Part A Appl. Sci. Manuf..

[52] Schartel B., Hull T.R. (2007). Development of fire-retarded materials—Interpretation of cone calorimeter data. Fire Mater..

[53] Ferry L., Dorez G., Taguet A., Otazaghine B., Lopez-Cuesta J.M. (2015). Chemical modification of lignin by phosphorus molecules to improve the fire behavior of polybutylene succinate. Polym. Degrad. Stab..

[54] Bacsa W.S., Ugarte D., Châtelain A., de Heer W.A. (1994). High-resolution electron microscopy and inelastic light scattering of purified multishelled carbon nanotubes. Phys. Rev. B.

[55] Li J., Li B., Zhang X., Su R. (2001). The study of flame retardants on thermal degradation and charring process of manchurian ash lignin in the condensed phase. Polym. Degrad. Stab..

[56] Liu L., Huang G., Song P., Yu Y., Fu S. (2016). Converting Industrial Alkali Lignin to Biobased Functional Additives for Improving Fire Behavior and Smoke Suppression of Polybutylene Succinate. ACS Sustain. Chem. Eng..

[57] Alongi J., Ciobanu M., Malucelli G. (2011). Novel flame retardant finishing systems for cotton fabrics based on phosphorus-containing compounds and silica derived from sol–gel processes. Carbohydr. Polym..

