# Supplementary material for: Development of Novel Polyamide 11 Multifilaments and Fabric Structures Based on Industrial Lignin and Zinc Phosphinate as Flame Retardants
Source: Molecules. 2020 Oct 27;25(21):4963. doi: 10.3390/molecules25214963 (PMC7663702; doi:10.3390/molecules25214963)
Supplement: Supplementary file 1 [file molecules-25-04963-s001.pdf]

Development of Novel Polyamide 11 Multifilaments and Fabric Structures Based on Industrial Lignin and Zinc Phosphinate as Flame Retardants

Neeraj Mandlekar [a,b c], *Aurélie Cayla* [a], *François Rault* [a], *Stéphane Giraud* [a], *Fabien Salaün* [a *], *Jinping Guan* [b]

[a] ENSAIT, GEMTEX – Laboratoire de Génie et Matériaux Textiles, F-59000 Lille, France;

[b] College of Textile and Clothing Engineering, Soochow

[c] Politecnico di Torino, Dept. of Applied Science and Technology, Alessandria, Italy

Table S1. Prepared blend formulations

Sample code	Polyamide 11 (wt%)	Lignosulphonate (wt%)	Domtar kraft lignin (wt%)	Zinc phosphinate (wt%)
PA11	100	-	-	-
PA80-LL20	80	20	-	-
PA80-KL20	80	-	20	-
PA80-ZnP20	80	-	-	20
PA-LL5-ZnP15	80	5	-	15
PA-LL7-ZnP13	80	7	-	13
PA-LL10-ZnP10	80	10	-	10
PA-KL5-ZnP15	80	-	5	15
PA-KL7-ZnP13	80	-	7	13
PA-KL10-ZnP10	80	-	10	10

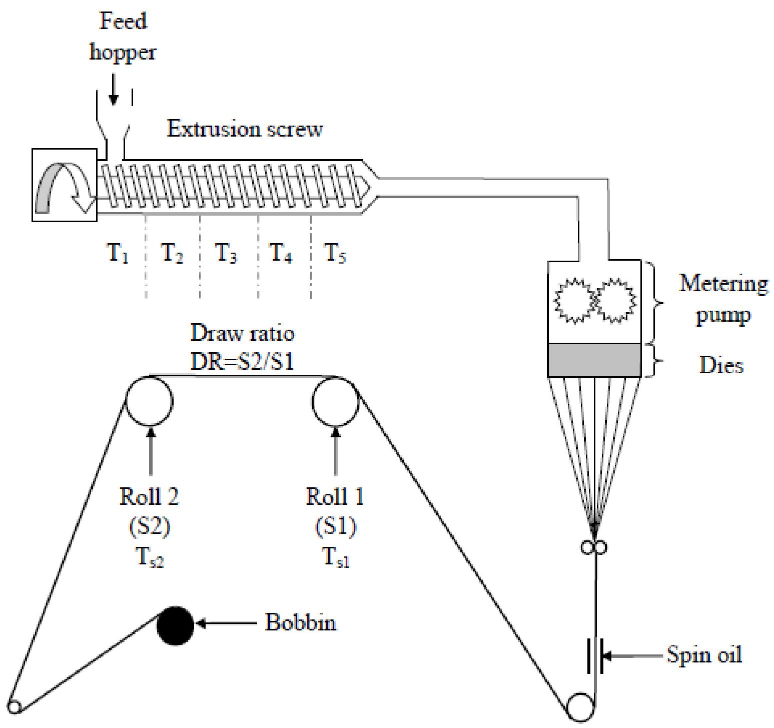

Figure S1. Schematic of melt spinning machine.

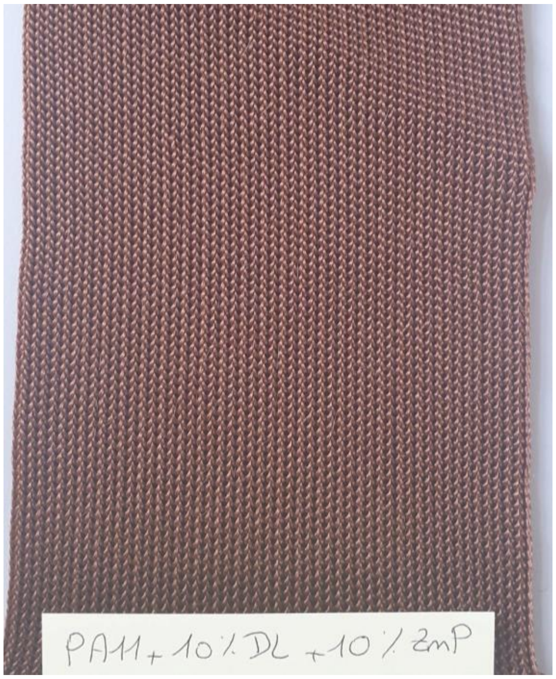

Figure S2. KL and ZnP filled PA11 knitted fabric structure.

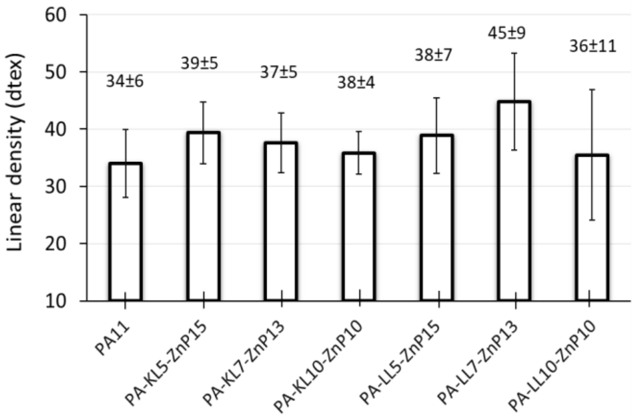

Figure S3. The linear density of monofilament yarns.
